# Recombinant human erythropoietin therapy in critically ill patients: a dose-response study [ISRCTN48523317]

**DOI:** 10.1186/cc3786

**Published:** 2005-08-05

**Authors:** Dimitris Georgopoulos, Dimitris Matamis, Christina Routsi, Argiris Michalopoulos, Nina Maggina, George Dimopoulos, Epaminondas Zakynthinos, George Nakos, George Thomopoulos, Kostas Mandragos, Alice Maniatis

**Affiliations:** 1 Professor of Medicine & ICU Director, Department of Intensive Care Medicine, University Hospital of Heraklion, University of Crete, Heraklion, Crete, Greece; 2 ICU Director, Intensive Care Unit, Papageorgiou Hospital of Thessaloniki, Thessaloniki, Greece; 3 Assistant Professor of Medicine, Department of Intensive Care, Evangelismos Hospital, University of Athens, Athens, Greece; 4 ICU Director, Intensive Care Unit, Henry Dunan Hospital, Athens; 5 ICU Director, Intensive Care Unit, Saint Olga Hospital, Athens, Greece; 6 Intensive Care Unit, Sotiria Hospital, Athens, Greece; 7 Assistant Professor of Medicine & ICU Director, Intensive Care Unit, University Hospital of Larisa, University of Larisa, Larisa, Greece; 8 Associate Professor of Medicine, Intensive Care Unit, University Hospital of Ioannina, University of Ioannina, Ioannina, Greece; 9 ICU Director, Intensive Care Unit, Laiko Hospital, Athens; 10 ICU Director, Hellenic Red Cross Hospital, Athens, Greece; 11 Professor of Medicine, University Hospital of Patras, Patras, Greece

## Abstract

**Introduction:**

The aim of this study was to assess the efficacy of two dosing schedules of recombinant human erythropoietin (rHuEPO) in increasing haematocrit (Hct) and haemoglobin (Hb) and reducing exposure to allogeneic red blood cell (RBC) transfusion in critically ill patients.

**Method:**

This was a prospective, randomized, multicentre trial. A total of 13 intensive care units participated, and a total of 148 patients who met eligibility criteria were enrolled. Patients were randomly assigned to receive intravenous iron saccharate alone (control group), intravenous iron saccharate and subcutaneous rHuEPO 40,000 units once per week (group A), or intravenous iron saccharate and subcutaneous rHuEPO 40,000 units three times per week (group B). rHuEPO was given for a minimum of 2 weeks or until discharge from the intensive care unit or death. The maximum duration of therapy was 3 weeks.

**Results:**

The cumulative number of RBC units transfused, the average numbers of RBC units transfused per patient and per transfused patient, the average volume of RBCs transfused per day, and the percentage of transfused patients were significantly higher in the control group than in groups A and B. No significant difference was observed between group A and B. The mean increases in Hct and Hb from baseline to final measurement were significantly greater in group B than in the control group. The mean increase in Hct was significantly greater in group B than in group A. The mean increase in Hct in group A was significantly greater than that in control individuals, whereas the mean increase in Hb did not differ significantly between the control group and group A.

**Conclusion:**

Administration of rHuEPO to critically ill patients significantly reduced the need for RBC transfusion. The magnitude of the reduction did not differ between the two dosing schedules, although there was a dose response for Hct and Hb to rHuEPO in these patients.

## Introduction

Anaemia is a common problem in critically ill patients [1,2]. Indeed, it has been shown that, at intensive care unit (ICU) admission, mean haemoglobin (Hb) concentration in critically ill patients is about 11 g/dl, and in 60% and 30% of them the mean Hb is less than 12 and 10 g/dl, respectively. Thus, the majority of critically ill patients exhibit anaemia upon ICU admission, which persists throughout the duration of their ICU stay. Overt or occult blood loss and decreased production of red blood cells (RBCs) due to blunted erythropoietic response are the two main causes of anaemia in these patients [3].

Anaemia in critically ill patients results in significant RBC transfusions. Approximately 40% of critically ill patients receive at least one unit of RBCs, relatively soon after ICU admission [1,2]. It is of interest that the mean number of RBC units transfused approaches five and the pretransfusion Hb is about 8.5 g/dl, indicating that the large number of transfusions is not due to a high transfusion threshold for Hb [1,2].

It has been recognized that RBC transfusion is not without risks. The adverse effects of RBC transfusions are numerous, including transmission of infection [4], transfusion associated immunosuppression [5-9], transfusion related acute lung injury [10], disturbances in microcirculation due to blood storage [11,12] and allergic reactions [9]. Large observational studies in critically ill patients have shown that RBC transfusion is an independent risk factor for increased mortality [1,2]. Although the mechanism through which RBC transfusion may increase mortality is currently unknown, studies have shown that RBC transfusion in critically ill patients is associated with a higher incidence of infection and evidence of tissue hypoxia [11,13,14]. These data indicate that the likely contributing factors to mortality are related to immunosuppression and disturbances in microcirculation, as opposed to allergic reaction or transmission of infection.

Because of the risks associated with blood transfusion, alternative treatments and preventative strategies for anaemia in critically ill patients have been explored. Among them, exogenous administration of recombinant human erythropoietin (rHuEPO) demonstrated promising results [15-17]. The rationale underlying therapy with rHuEPO therapy in critically ill patients is that increased erythropoiesis will result in higher Hb levels and subsequently reduce the need for RBC transfusion [18]. It has been shown that exogenous rHuEPO in critically ill patients raised reticulocyte counts and Hb, and reduced considerably requirements for RBC transfusion [15-17].

The two randomized studies that showed that rHuEPO is efficacious in increasing Hb level and reducing allogeneic RBC transfusion used two different therapeutic regimens [15,16]. One study [16] used 300 units/kg rHuEPO for 5 consecutive days and then every other day to achieve a haematocrit (Hct) concentration above 38%, whereas in the other [15] the drug was administered weekly in a dose of 40,000 units. Thus, the optimal dose of rHuEPO in critically ill patients is not known, which is an issue of financial importance, given the cost of this therapy.

The aim of the present study was to assess the efficacy of two dosing schedules of rHuEPO (40,000 units once and thrice per week, respectively) in increasing Hct and Hb and in reducing exposure to allogeneic RBC transfusion in critically ill patients. These dosing regimens are comparable to those used by the two randomized studies in critically ill patients [15,16].

## Materials and methods

This study was a prospective, randomized, multicentre trial conducted at 13 Greek ICUs between November 2000 and December 2003. Approval of the study was given by the institutional review committee at each participation centre and written informed consent was obtained from each patient or next of kin. Patient enrollment was done at each site and supervised by the data coordinating centre. Randomization and data analysis were done by the data coordinating centre. A stratified random sampling scheme was employed as the selection method for randomization. Acute Physiology and Chronic Health Evaluation II score and age decades were considered as distinct strata. To ensure equal allocation of individuals from each stratum (epsem scheme), the sampling fraction was considered. The sample size was calculated in order to detect a 10% difference in the Hct values between groups receiving rHuEPO at a 5% significance level and 90% power, assuming that the mean Hct for the group receiving the lowest dose would be in the range of 35% and the variance equal to 30.

All patients admitted to the ICU in each of the 13 participating centres were evaluated for study eligibility. Inclusion criteria were as follows: age at least 18 years; Hb less than 12 g/dl; no iron deficiency (defined as transferrin saturation <10% and ferritin <50 ng/ml); negative pregnancy test (for females of reproductive age); an expected ICU stay of at least 7 days; and provision of signed informed consent. The expected duration of the ICU stay was judged on clinical grounds and Acute Physiology and Chronic Health Evaluation II score by the ICU team at admittance to the unit. Exclusion criteria included chronic renal failure requiring dialysis, new onset (<6 months) seizures, life expectancy under 7 days, previous use of rHuEPO (within 3 months), recent use of cytostatics or recent radiotherapy (within 1 month) and participation in another research protocol.

The patients were randomly assigned (day 0) to receive intravenous iron saccharate alone (control group), intravenous iron saccharate and subcutaneous rHuEPO 40,000 units once per week (group A), and intravenous iron saccharate and subcutaneous rHuEPO 40,000 units three times per week (group B). In all groups iron was given at a dose of 100 mg three times per week. rHuEPO was provided by the sponsor of the trial.

rHuEPO was given for a minimum of 2 weeks or until ICU discharge or death. The maximum duration of therapy was 3 weeks. rHuEPO was temporarily withheld when Hb exceeded 12 g/dl and was resumed if Hb again fell to below 12 g/dl. rHuEPO was given intravenously if the platelet count was below 20,000 μL.

Transfusion of RBCs was standardized at a Hb of 7 g/dl and, in cases of active cardiac ischaemia and central nervous system damage, at 9 g/dl [19]. In patients with active blood loss, defined as evidence of ongoing blood loss accompanied by a decrease in the Hb concentration of 3.0 g/dl in the preceding 12 hours or a requirement for at least 3 units of packed RBCs during the same period, the need for blood transfusion was determined by the patient's attending physician. The physicians caring for the patients were instructed to administer RBC transfusions, one unit at a time, and to measure the patient's Hb concentration after each unit was transfused.

The primary outcome end-points were differences in Hct and Hb between groups and transfusion independence between study days 1 and 28. Additional data recorded included ICU length of stay and cumulative mortality through to day 28. Adverse effects were assessed daily. Nosocomial infections were diagnosed using standard criteria [20,21].

All patients were followed up for a total of 28 days from the day of randomization, unless death occurred earlier. Patients discharged from the hospital before study day 28 had final laboratory data obtained within 5 days of study day 28. Patients were followed up for 28 days, unless death occurred earlier. Patients who were discharged from the hospital before study day 28 and were not available to provide the final laboratory data (i.e. data were not available within 5 days of study day 28) were considered lost to follow up. Analysis of outcomes was on an intent-to-treat basis.

All categorical variables were summarized by frequency distribution tables and analyzed by χ^2 ^tests. Descriptive results for continuous measurements were presented as mean ± standard deviation unless otherwise stated. The methods used for analysis were analysis of variance F tests, Scheffe tests for multiple comparisons, Kruskal–Wallis and Mann–Whitney tests where appropriate. The transfusion rate was analyzed using a zero-inflated Poisson model. All computations were done using Sigmastat-plus (SPSS, INC, Chicago, Ill). All statistical tests were two-sided, and the level of statistical significance was set at 5%.

Patients who did not receive a transfusion at the time of study withdrawal, who died, or who were lost to follow up after hospital discharge were considered nontransfused for the analysis. The number of RBC units transfused, transfusion rate, average days transfused and units per transfused patient were analysed using the Mann–Whitney test. Transfusion rate, expressed as the number of RBC units transfused per day during the study, was determined by dividing the number of transfused units for each group by the total number of days alive for the patients in the group. Average days transfused was determined by dividing the number of transfusion days for each group by the total number of days alive for the patients in the group. The average number of units transfused was determined by dividing the number of transfusions for each group by the total number of patients in the group. Units per transfused patient were determined by dividing the number of transfusions for each group by the total number of patients transfused in the group.

## Results

**Figure 1:**
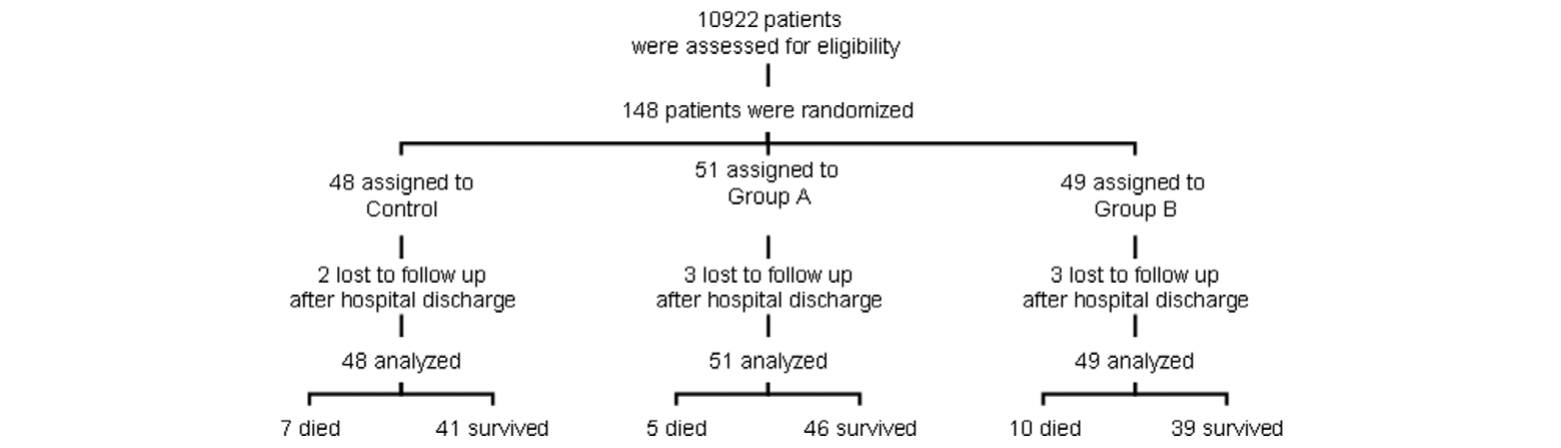
Study flow chart.

A total of 148 patients were enrolled in the study (Fig. 1). Forty-eight patients were randomly assigned to the control group, 51 to group A (40,000 units of rHuEPO once per week) and 49 to group B (40,000 units of rHuEPO three times per week). At baseline the demographic characteristics and severity of the disease did not differ significantly between groups (Table 1). All patients were mechanically ventilated at the time of enrollment. This was because the attending physicians did not expect an ICU stay to exceed 7 days if the patient did not need mechanical ventilatory support at the time of randomization.

The pretransfusion Hct and Hb did not differ significantly between groups, averaging 24.5 ± 3.2%, 24.1 ± 2.7% and 23.5 ± 1.8%, and 7.9 ± 1.1 g/dl, 7.6 ± 0.8 g/dl and 7.7 ± 0.9 g/dl, respectively, in the control group, group A and group B. The cumulative number of RBC units transfused, the average RBC units transfused per patient and per transfused patient, and the average volume of RBC transfused per day were significantly higher in the control group than in groups A and B, whereas the differences between groups A and B were not significant. Also, the percentage of transfused patients was significantly higher in control group than in groups A and B (Table 2). Noncompliance of physicians with the transfusion strategy, as indicated by a finding of pretransfusion Hb 0.5 g/dl higher than the transfusion threshold, occurred on 10 occasions in control group (7% of the total units transfused in control group), on seven in group A (21%) and on three (13%) in group B (*P *> 0.05).

Transfusion rate represents the mean transfusion per patient per day. Because of the presence of many zeros, a zero-inflated Poisson distribution was deemed suitable for modelling the data [22]. This is approximately equivalent to using two separate analyses. The first is the percentage of patients with no transfusion requirement and the second is the fit of a Poisson regression to the data for the transfused patients only. The percentages of patients with no need for transfusion were 41.7%, 62.7% and 73.5% for the control group, group A and group B, respectively. The percentage in group B was statistically different from that in the control group (Fisher's exact test with Bonferroni correction, *P *= 0.002). The percentage in group A did not differ significantly from those in group B and the control group. Considering the transfusion rate for the transfused patients only, group A exhibited the lowest value (22.8 ml). This value was significantly different from the corresponding transfusion rates for patients of group B (32.5 ml; *P *< 0.001) and the control group (59.3 ml; *P *< 0.001).

There was a dose response of Hb and Hct to rHuEPO, which was evident from study days 14 to 28 (Table 3). The mean increase in Hct (ΔHct) and Hb (ΔHb) from baseline to the final measurement was significantly greater in group B than in the control group (Table 2). ΔHct was significantly higher in group B than in group A. ΔHct in group A was significantly higher than in control individuals, whereas ΔHb did not differ significantly between the control group and group A (Table 2).

There was no significant difference in lengths of ICU and hospital stay among the three groups. Mean ICU length of stay averaged 21.8 ± 8.2, 21.0 ± 8.3 and 19.6 ± 8.8 days in the control group, group A and group B, respectively (*P *> 0.05). Seven patients stayed in the ICU for less than 7 days, two of whom were in the control group and five were in group B. Exclusion of these patients did not materially alter the results (22.5 ± 7.4, 21.0 ± 8.3 and 21.3 ± 7.5 days, respectively, in the control group, group A and group B). There was a weak relationship between the total transfusion need (in ml) and length of ICU stay (*r *= 0.162, *P *= 0.05). Again, exclusion of the seven patients who stayed in the ICU for less than 7 days did not change that relationship. Also, mean ICU free days did not differ between groups, averaging 6.3 ± 8.2 days in the control group, 7.0 ± 8.3 days in group A and 8.5 ± 8.8 days in group B. There was no significant difference in mean ventilator free days among groups (10.3 ± 10.6 days in the control group, 11.1 ± 11.5 days in group A and 11.9 ± 10.4 days in group B).

Seven, five and ten patients died, respectively, in the control group, group A and group B, resulting in corresponding mortality rates of 14.6%, 9.8% and 20.4% (*P *> 0.05). The incidence of serious adverse events reported was comparable between the three groups (Table 4). At least one adverse event occurred in 23 patients (48.8%) in the control group, in 21 (41.2%) in group A and in 22 (45.8%) in group B.

## Discussion

The main findings of our study were as follows: in critically ill patients rHuEPO administration significantly reduced the need for RBC transfusions; the magnitude of this reduction did not differ between the two dosing schedules; and there was a dose response of Hct and Hb to rHuEPO in these patients.

The study was not blinded and this might be a limitation. The designers of the study considered it unethical to administer placebo subcutaneously to critically ill patients. However, we believe that this limitation is unlikely to have influenced the results. Contrary to other studies dealing with rHuEPO administration in critically ill patients [15,16], in our study a specific transfusion threshold was applied. Indeed, the indications for transfusion were predefined and based on objective indices [19]. This is further supported by the similar value of pretransfusion Hb and Hct in the three groups of patients, indicating that our results cannot be accounted for by different transfusion strategies between groups. In addition, the rate of failure of physicians to adhere to the predefined transfusion trigger was comparable between the three groups. These factors suggest that the lack of blinding was not a significant confounding factor.

In both dosing regimens rHuEPO was given in much higher doses than are used in patients who are not critically ill [23]. However, it is well known that the requirement for erythropoietin is increased in patients with severe illness [24]. In addition, the studies that found an effect of rHuEPO on erythropoiesis in critically ill patients [15-17] used doses in the range of 40,000 to approximately 120,000 units per week, which are comparable to those given in our study.

In our study 58% of patients randomly assigned to the control group received transfusion, and on average they received approximately 5 units of RBCs. These findings are remarkably similar to those reported by other studies [1,2] and emphasize the great number of ICU patients who need RBC transfusion. We also showed that exogenous administration of rHuEPO to ICU patients at a dose of 40,000 units once or thrice per week was able to reduce the number of transfused patients by 36% and 55%, respectively. Similar results were also reported by two randomized studies showing that rHuEPO at a dose of 300 units/kg for 5 days followed by administration every other day [15] or 40,000 once weekly [16] decreased the number of transfused critically ill patients by approximately 18%. We further showed that the reduction in the proportion of transfused patients, the total and daily RBC units transfused, the transfused RBC units per patient and per transfused patient, and the average days transfused did not differ between the two dosing regimens. The similar reduction in the need of transfusion indicates that if the purpose of the administration of rHuEPO is to reduce RBC transfusion in critically ill patients, then 40,000 once weekly is probably sufficient. Higher doses increase the cost of therapy considerably, whereas on the other hand they are unlikely to have significant impact on transfusion needs. However, we should note that these results were obtained with predefined indications for transfusion based on a certain restrictive transfusion strategy that is currently recommended [19]. Different results might have been obtained if other transfusion strategies were used.

It is believed that critically ill patients have limited ability to compensate for the fall in Hb concentration [25,26]. Indeed, in these patients anaemia is associated with increased morbidity and mortality, particularly in patients with pre-existing cardiac disease [25,26]. Transfusion of RBCs may not be the ideal therapy for these patients because there is a growing body of evidence indicating that RBC transfusion in critically ill patients is an independent risk factor for increased morbidity and mortality [1,2]. In addition, it has been shown that in the majority of critically ill patients the transfused blood is relatively old (>10 days) [2] and this may limit the ability of transfused RBCs to increase the supply of oxygen to tissues [11]. The immunomodulatory effects of blood transfusion is also of great concern in critically ill patients in whom the risk for infections is high. It was recently shown in patients undergoing hip replacements that the levels of natural killer cell precursors and interferon-γ were substantially reduced by the surgery and blood loss and by transfusions of allogeneic nonleucodepleted, allogeneic leucodepleted, and autologous predeposit blood [5]. Considering that critically ill patients are often immunoparalyzed [27], the immunosuppressive effects of blood transfusion should be taken into account. Thus, preventing anaemia by administration of rHuEPO minimizes the risks for anaemia but without exposing the critically ill to the deleterious effect of RBC transfusion. Studies have shown that rHuEPO can achieve this goal [15-17]. We further demonstrated a dose response of Hct and Hb to rHuEPO; Hct and Hb increased with increasing dose of rHuEPO. It is of interest that the final values of Hb and Hct achieved with the highest dose of rHuEPO were close to normal. It follows that if the goal of rHuEPO therapy is to attain normal values of Hct and Hb, then 40,000 units thrice per week may be a reasonable strategy.

Corwin and coworkers [28] showed that administration of rHuEPO in critically ill patients at a dose of 40,000 once per week was not associated with increased side effects. These findings are further extended by our study, demonstrating that rHuEPO even at higher doses of 40,000 three times per week is probably safe; no significant difference was observed between rHuEPO groups and the control group in terms of side effects. Nevertheless, the sample size was relatively small, and so comments regarding safety should be made with great caution.

It is currently unclear whether administration of rHuEPO in critically ill patients is associated with improved outcome. Our study was underpowered to demonstrate an effect of rHuEPO on mortality or resource utilization (i.e. length of ICU or hospital stay, and ICU and ventilator free days). Nevertheless, Corwin and coworkers [28] reported that neither morbidity nor mortality differed significantly between critically ill patients receiving rHuEPO 40,000 once per week and a placebo group. However, interpretation of these results is complicated by the fact that the majority of patients receiving rHuEPO were anaemic by the end of the study, and Hb level differed slightly between groups (approximately 0.3 g/dl). Considering the relationship between the level of anaemia and morbidity and mortality [25,26], the inability of this rHuEPO regimen to increase Hb to normal levels might have influenced the morbidity and mortality data. Perhaps doses of rHuEPO that result in near normal values of Hb and Hct such as those used in the present study (i.e. 40,000 units three times per week) may be associated with improved outcome. Further studies with the appropriate power are needed to resolve this issue.

Finally, we should emphasize that the present study was designed to evaluate the efficiency of two dosing regimens of rHuEPO in increasing Hb and Hct and decreasing transfusion requirements, and not to collect pharmacoeconomic data. Although the study provides some data such as length of stay and pharmaceutical treatment, it is very difficult to estimate precisely the cost of overall medical services because Greek National Health System prices are underestimated because of the fact that the System is based on very low charges for patients, largely subsidized by taxation.

## Conclusion

In conclusion, the present study showed that administration of rHuEPO reduces the need for RBC transfusion in critically ill patients. The magnitude of this reduction was similar between the two dosing schedules. On the other hand, rHuEPO increased Hb and Hct in a dose dependent manner. These results indicate that dose of rHuEPO in critically ill patients should be titrated depending on the desired goal.

## Key messages

• 	Administration of rHuEPO reduced the need for RBC transfusion in critically ill patients.

• 	The magnitude of this reduction was similar between the two dosing schedules.

• 	rHuEPO administration increases Hb and Hct in a dose dependent manner.

• 	The dose of rHuEPO in critically ill patients may be titrated depending on the desired goal.

## Abbreviations

Hb = haemoglobin; Hct = haematocrit; ICU = intensive care unit; RBC = red blood cell; rHuEPO = recombinant human erythropoietin.

## Competing interests

The author(s) declare that they have no competing interests.

## Authors' contributions

DG designed the study, supervised the study, analyzed the data and wrote the manuscript. DM, CR, AM, AM, GD, EZ, GN, GT, KM and AM designed the study.

## Figures and Tables

**Table 1:** Demographics and baseline characteristics (at day 0)

Characteristic/parameter	Control group	Group A	Group B
Number of patients	48	51	49
Age (years [median, range])	58 (19–86)	60 (19–91)	63 (22–88)
Sex (*n*)			
Men	33	41	39
Women	15	10	10
APACHE II score (mean ± SD)	14.4 ± 6.1	14.4 ± 5.3	14.9 ± 5.9
Admitting diagnosis (*n*)			
Trauma	12	19	20
Surgical	9	6	11
Nonsurgical	27	26	18
Baseline laboratory values (day 0)			
Hct (% [mean ± SD])	28.3 ± 3.7	28.2 ± 3.7	28.4 ± 2.8
Hb (g/dl [mean ± SD])	9.2 ± 1.3	9.3 ± 0.9	9.3 ± 1.2
Reticulocytes (% [mean ± SD])	3.0 ± 4.2	3.1 ± 3.2	3.6 ± 4.4
Iron (μg/dl [mean ± SD])	41.3 ± 23.0	46.2 ± 37.1	39.7 ± 24.4
Ferritin (ng/dl [mean ± SD])	590.6 ± 454.3	453.5 ± 443.1	561.3 ± 489.5
Transferrin saturation (% [mean ± SD])	23.7 ± 13.1	22.3 ± 12.6	24.8 ± 12.7

The three groups were comparable at enrollment (*P *> 0.05) with respect to baseline demographic characteristics, admitting diagnosis, severity score and laboratory values. APACHE, Acute Physiology and Chronic Health Evaluation; Hct, haematocrit; Hb, haemoglobin; SD, standard deviation.

**Table 2:** Study outcomes

Parameter	Control	Group A	Group B
Total units transfused	138	33*	23*
% of transfused patients	58.3	37.3*	26.5*
Units transfused per patient	2.83 ± 3.9	0.64 ± 1.0*	0.47 ± 0.9*
Units transfused per transfused patient	4.86 ± 4.0	1.74 ± 0.7*	1.77 ± 0.7*
Volume of RBCs transfused per day	43.2 ± 61.1	11.3 ± 20.3*	16.1 ± 36.1*
Average days transfused	1.60 ± 2.2	0.59 ± 0.9*	0.35 ± 0.7*
Hct (%) at day 28	30.4 ± 4.5	33.3 ± 5.3*	37.5 ± 5.8* ^†^
Hb (mg/dl) at day 28	9.9 ± 1.5	10.7 ± 1.9	11.6 ± 1.9*
ΔHct (%)	2.09 ± 5.0	5.53 ± 5.5*	8.76 ± 6.2* ^†^
ΔHb (mg/dl)	0.69 ± 1.5	1.43 ± 1.7	2.24 ± 6.2*

*P < 0.05 versus control; ^†^*P *< 0.05 versus group A. ΔHb, mean increase in Hb from baseline to final measurement; ΔHct, mean increase in Hct from baseline to final measurement; Hb, haemoglobin; Hct, haematocrit; RBC, red blood cell.

**Table 3:** Haematocrit and haemoglobin on different study days

Day	Haematocrit			Haemoglobin		
	
	Control	Group A	Group B	Control	Group A	Group B
0	28.3 ± 3.7	28.2 ± 3.7	28.4 ± 2.8	9.2 ± 1.3	9.3 ± 1.2	9.2 ± 0.9
3	27.9 ± 3.7	26.7 ± 5.0	28.2 ± 3.7	9.1 ± 1.3	9.0 ± 1.1	9.1 ± 1.2
7	28.4 ± 4.1	28.4 ± 3.7	29.5 ± 4.0	9.1 ± 1.4	9.2 ± 1.2	9.5 ± 1.3
10	28.8 ± 4.5	29.4 ± 4.1	30.3 ± 4.2*	9.4 ± 1.6	9.6 ± 1.2	9.7 ± 1.4
14	28.8 ± 4.5	30.8 ± 4.5	32.3 ± 5.4*	9.4 ± 1.4	10.0 ± 1.5	10.3 ± 1.9*
21	29.4 ± 6.2	31.9 ± 5.0	36.5 ± 6.3* ^†^	9.7 ± 2.0	10.3 ± 1.7	11.3 ± 2.1*
28	30.4 ± 4.5	33.3 ± 5.3	37.5 ± 5.8* ^†^	9.9 ± 1.5	10.6 ± 1.9	11.6 ± 1.9*

Shown are mean ± standard deviation values for haematocrit and haemoglobin on different study days. *P < 0.05 versus control; ^†^*P *< 0.05 versus group A.

**Table 4:** Serious adverse events

Adverse event	Control	Group A	Group B
Deaths	7	5	10
Nosocomial infection	21	22	26
Heart rate and rhythm disorders	3	2	2
Thrombocytosis	1	-	1
Deep vein thrombosis	2	-	1
Central nervous system disorders (new ischaemic or haemorrhagic stroke)	3	2	2
